# Factors Predicting HBsAg Seroclearance and Alanine Transaminase Elevation in HBeAg-Negative Hepatitis B Virus-Infected Patients with Persistently Normal Liver Function

**DOI:** 10.1371/journal.pone.0166543

**Published:** 2016-12-09

**Authors:** Tai-Long Chien, Jing-Houng Wang, Kwong-Ming Kee, Chien-Hung Chen, Chao-Hung Hung, Sheng-Nan Lu

**Affiliations:** 1 Division of Hepato-Gastroenterology, Department of Internal Medicine, Antai Medical Care Cooperation Antai Tian-Sheng Memorial Hospital, Donggang Township, Pingtung County, Taiwan; 2 Division of Hepato-Gastroenterology, Department of Internal Medicine, Kaohsiung Chang Gung Memorial Hospital and Chang Gung University College of Medicine, Kaohsiung City, Taiwan; Universita degli Studi di Pisa, ITALY

## Abstract

**Background:**

A certain proportion of hepatitis B virus (HBV)-infected patients with persistently normal alanine transaminase (ALT) levels have significant fibrosis. Using liver stiffness measurements (Fibroscan^®^) and laboratory data, including serum ALT, quantitative HBsAg (qHBsAg), and HBV DNA, we attempted to predict the natural histories of these patients.

**Methods:**

Non-cirrhotic HBeAg-negative chronic hepatitis B patients with persistently normal ALT were followed up prospectively with the end points of HBsAg seroclearance and ALT elevation above the upper limit of normal. The factors that were predictive of the end points were identified.

**Results:**

A total of 235 patients with an average age of 48.1 +/- 10.7 years were followed up for 7 years. Eight patients (3.4%) lost HBsAg, and 15 patients (6.4%) experienced ALT elevation. The overall cumulative HBsAg seroclearances were 0.4%, 1.3% and 2.3% at years 1, 3 and 5, respectively. Regarding HBsAg seroclearance, the qHBsAg (< 30 IU/ml) cutoff resulted in a hazard ratio (HR) of 19.6 with a 95% confidence interval (CI) of 2.2–166.7 (P = 0.008). The baseline ALT level (odd ratio (OR) 1.075, 95% CI 1.020–1.132, P = 0.006) and a qHBsAg above 1000 IU/ml (3.7, 1.1–12.4, P = 0.032) were associated with ALT elevation. Limited to men, the baseline liver stiffness (1.6, 1.0–2.5, P = 0.031) and a qHBsAg above 1000 IU/ml (10.4, 2.1–52.4, P = 0.004) were factors that were independently associated with ALT elevation.

**Conclusion:**

A low qHBsAg level predicted HBsAg clearance. Baseline ALT and a qHBsAg above 1000 IU/ml were independent predictive factors for ALT elevation. Among the men, the independent predictive factors for ALT elevation were qHBsAg and liver stiffness.

## Introduction

Most of the treatment guidelines suggest that chronic hepatitis B (CHB) patients with persistently normal alanine aminotransferase (ALT) levels should not undergo antiviral therapy with the exceptions of liver cirrhosis patients and patients with liver biopsies that exhibit significant fibrosis. Previous studies have demonstrated that high serum HBV DNA levels increase the risks of liver cirrhosis and/or hepatocellular carcinoma (HCC). A certain proportion of patients with normal ALT levels have significant inflammation or fibrosis [1,2]. Most of the treatment guidelines suggest that chronic hepatitis B (CHB) patients with persistently normal alanine aminotransferase (ALT) levels should not undergo antiviral therapy with the exceptions of liver cirrhosis patients and patients with liver biopsies that exhibit significant fibrosis. Previous studies have demonstrated that high serum HBV DNA levels increase the risks of liver cirrhosis and/or hepatocellular carcinoma (HCC). A certain proportion of patients with normal ALT levels have significant inflammation or fibrosis [1,2]. One report found that among the patients with ages older than 40 years and ALT levels in the high normal range, up to 47% of patients with persistently normal ALT and an HBV DNA value > 10,000 copies/ml exhibit significant fibrosis and inflammation on liver biopsy. In contrast, the other two studies showed that most of the patients in the immune tolerant phase of chronic HBV infection exhibited no or minimal fibrosis despite high HBV DNA levels [3,4]. These data indicate that duration of infection and age are important for assessing the severity of liver injuries in patients with high HBV DNA but normal ALT levels. In recent years, the quantification of HBsAg has become an important factor in the evaluation of viral activity. Brunetto et al. [5] first studied the relationship between the serum HBsAg levels and the clinical stages of HBeAg-negative HBV carriers with genotype D infections. They found that a combination of single measurement HBV DNA (< 2000 IU/ml) and quantification of HBsAg (< 1000 IU/ml) could identify inactive carriers with 94.3% diagnostic accuracy [5]. Tseng et al. [6] reported that risks of HBeAg-negative hepatitis, hepatitis flares, and cirrhosis were low in patients with qHBsAg (< 1000 IU/ml) [6]. Lower levels of both quantitative HBsAg and HBV DNA might represent an inactive HBV infection [7,8]. Hepatic fibrosis indicates accumulated liver damage and is also a prognostic factor for chronic hepatitis B disease. A non-invasive method, such as transient elastography, makes the measurement of fibrosis easy and available. In this study, we used the baseline ALT and HBV DNA levels, the qHBsAg levels and Fibroscan^®^ results to predict the natural courses of HBeAg negative hepatitis B virus-infected patients with persistently normal ALT.

## Patients and Methods

### Study populations

All the HBsAg tests from 1999 to 2010 were searched in our computerized database of central laboratories. HBeAg-negative patients were enrolled. Those aged over 20 with persistent normal ALT for more than 1 year before entering the study and positive HBsAg and negative A-HCV statuses during a 2-year period and who were subjected to regular follow-up by four hepatologists were enrolled in this prospective study. Each patient was followed at 6-month intervals over 2 years before entering the study to ensure that the liver enzymes were within the normal limits. HBV-related test results were identified for 130,306 patients, and positive HBsAg positivity was noted in 24,182 patients. A total of 984 person-times from the 33,768 person-times satisfied the conditions. Two hundred ninety-three patients signed the forms and joined the study. Fifty-eight patients were excluded due to HBeAg positivity, liver cirrhosis and loss to follow-up. A total of 235 HBeAg-negative patients were enrolled in this study. Our Institutional Review Board approved the study.

### Follow-up

These 235 patients were followed from November 2007 to November 2014. The exclusion criteria included HBeAg positivity, liver cirrhosis based on ultrasound, co-infection with HCV or HIV, hemodialysis, chemotherapy and transplantation. Pregnant and breastfeeding women were also excluded. At the initial visit, the following laboratory data were collected: hemoglobin level, platelet count, aspartate transaminase (AST), ALT, alpha fetoprotein (AFP), total bilirubin, quantitative HBsAg (qHBsAg), hepatitis B virus DNA (HBV DNA), Fibroscan results, lipid profile and body mass index (BMI). The patients were followed up on a 6- or 12-month basis.

### Laboratory methods

#### Serology study

The following parameters were measured: HBsAg (MEIA, Abbott, North Chicago, IL, or Elecsys HBsAgII, Roche Diagnostics GmbH, Mannheim Germany), Roche Diagnostics GmbH (Mannheim, Germany), quantitative HBsAg level (HBs Ag quantitative kit (Abbott, North Chicago), AST (ULN: 37 U/L with GOT-L-J2 reagent, WAKO, Japan), ALT (ULN: 40 U/L; by GPT-L-J2 reagent, WAKO, Japan.), CBC (with a Sysmex XE-5000 Hematology Analyzer KOB, Koba, Japan), and AFP (with an ARCHITECT AFP assay; Abbott Ireland). The serum HBV DNA was quantified using TaqMan HBV analyte-specific reagent (ASR; Roche Molecular Systems, Inc., Branchburg, New Jersey, USA), which has a detection limit of 70 copies/ml.

#### Liver stiffness measurements

A one-dimensional transient elastography technique applied with the Fibroscan^®^ (Echosens, Paris, France) system was used for the liver stiffness measurements. During the examinations, the patients were lying on their backs with their right arms raised and tucked behind their head. A staff member placed the probe against the skin (in an intercostal space) and located a suitable portion of the liver of at least 6 cm in thickness that was free of large vascular structures. The acceptable range of the measurement depth was between 2.5 cm and 6.5 cm. Ten successful measurements were required for reliable results [9].

#### Statistical analysis

All data were recorded and analyzed using the paired t-test, chi-square test, Cox regression analysis, the Kaplan–Meier survival curve with the log-rank test and binary logistic regression for the analyses. The results are expressed as the means ± the standard deviations (SDs). A ROC analysis was performed to define the most suitable quantitative HBsAg value based on Yuden’s index (max [sensitivity +specificity-1]). The statistical procedures were performed with the SPSS statistical software version 17. P values < 0.05 were considered significant.

#### Ethics

Every patient agreed and signed the informed consent. The local institutional review board in Chang Gung Memorial Hospital approved the study.

## Results

**Fig 1:**
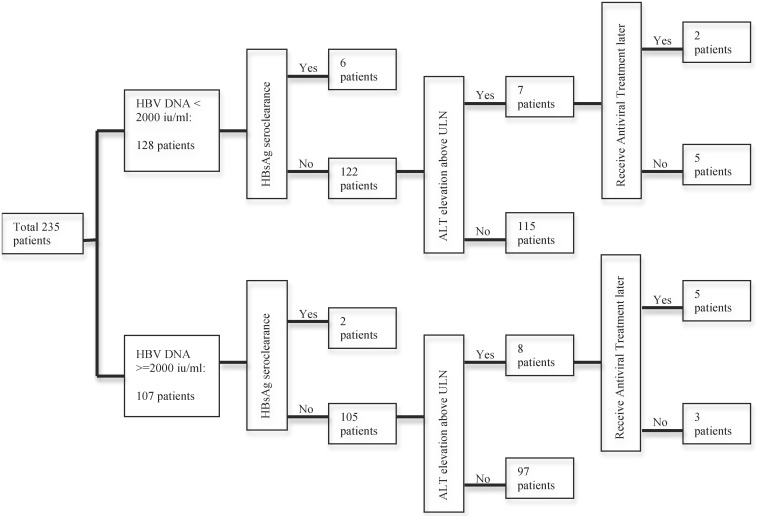
Natural histories of HBeAg-negative hepatitis B patients with normal liver function.

A total of 235 HBeAg-negative patients including 131 men and 104 women were enrolled. The endpoints were HBsAg seroclearance and ALT elevation (i.e., at least one episode of >40 u/l (the upper limit of normal, ULN) during the follow-up period. Eight patients (3.4%) exhibited HBsAg seroclearance, 15 patients (6.4%) experienced ALT elevation, and 212 patients (90.2%) did not exhibit HBsAg seroclearance or ALT elevation (Fig 1).

### HBsAg seroclearance

**Fig 2:**
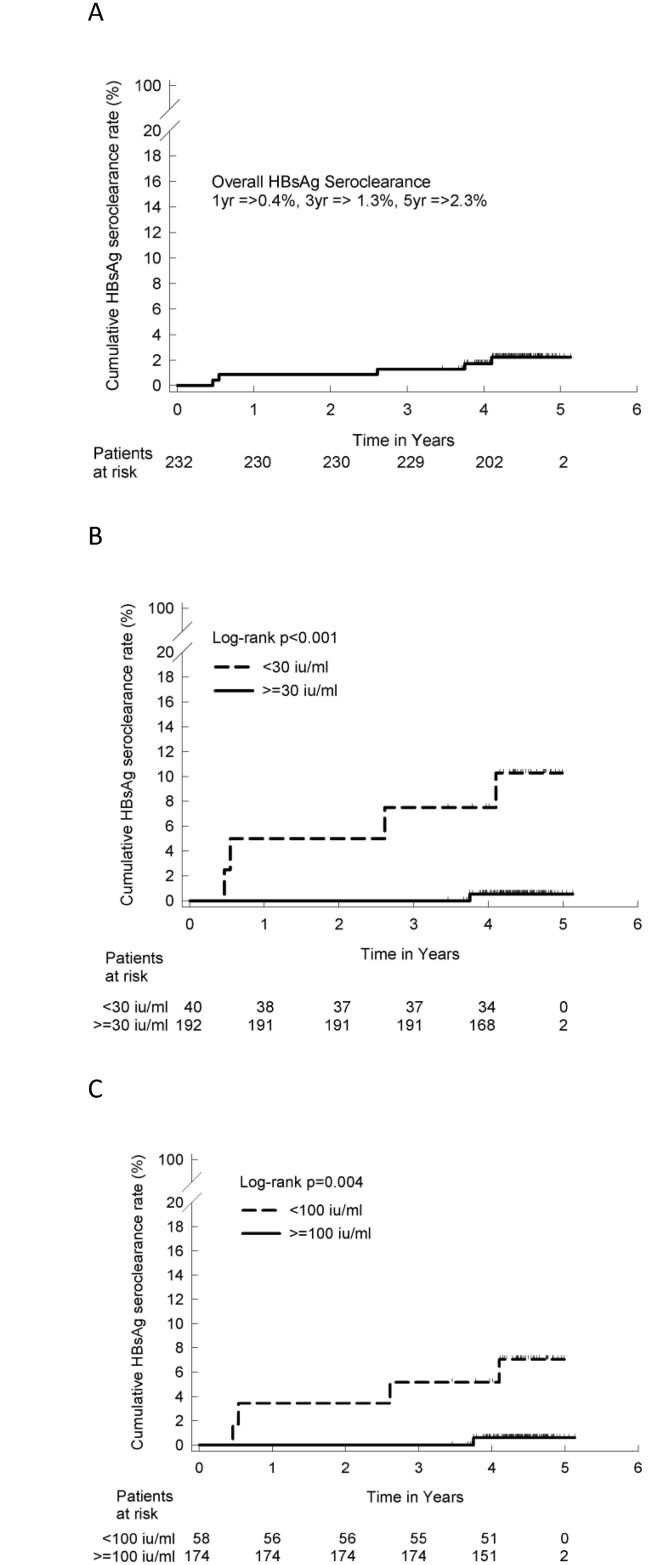
Relationship between quantitative HBsAg (qHBsAg) and the rate of hepatitis B surface antigen (HBsAg) seroclearance in the natural courses of the HBeAg-negative patients. Overall Cumulative HBsAg seroclearance (A), HBsAg seroclearance with a qHBsAg cutoff value of 30 IU/ml (B), and HBsAg seroclearance with a qHBsAg cutoff value of 100 IU/ml (C). The patients were stratified based on the baseline qHBsAg status (B, C).

Among the 8 patients who exhibited HBsAg seroclearance, the HBsAg data at the entry point were lost; thus, we used the data from the remaining 5 patients for the statistical analysis (Table 1). The most important factor for predicting HBsAg seroclearance was the qHBsAg. The median age of the HBsAg seroclearance patients was 51.6 years +/- 7.5 years. The cutoff point was identified via a ROC analysis. The area under the curve was 0.835. The best cutoff points were 28.9 IU/ml (in this study, we set the cutoff at 30 IU/ml, which yielded a sensitivity and specificity of 80% and 84%, respectively) and 97.5 IU/ml (in this study, we set the cutoff at 100 IU/ml, which yielded a sensitivity and specificity of 80% and 76%, respectively). The overall cumulative proportions of HBsAg seroclearance patients were 0.4%, 1.3% and 2.3 at years 1, 3 and 5, respectively (Fig 2A). Using a qHBsAg cutoff point of 30 IU/ml, the HBsAg seroclearance rates were 5%, 7.5% and 10.3% at years 1, 3 and 5, respectively (Fig 2B). Using an HBsAg cutoff point of 100 IU/ml, the HBsAg seroclearance rates were 3.4%, 5.2% and 7.1% at years 1, 3 and 5, respectively (Fig 2C). The hazard ratio (HR) for the 30 IU/ml qHBsAg cutoff was 19.6 with a 95% confidence interval (CI) of 2.2–166.7 (P = 0.008, Table 2).

**Table 1:** Basic characteristics of the HBsAg seroclearance and non-seroclearance patients.

Group	HBsAg Seroclearance	HBsAg Non-Seroclearance	P value*
Patient Number	5	227	
Age (years)	51.6±7.5	47.9±10.8	0.445
Sex (Male:Female)	4:01	102:125	0.267
Genotype (B, C, unknown)	3:02:00	168:47:12	0.533
DM (%)	0 (0%)	8 (3.5%)	0.589
Hypertension (%)	0(0%)	28(12.3%)	0.29
BMI (kg/m2) (no. 5–225)	23.5±3	23.3±3.2	0.933
Total cholesterol (mg/dl)	184.7±15.5	189.8±33.6	0.463
Triglyceride (mg/dl)	146.7±119.3	96.6±58.4	0.764
HDL (mg/dl)	50.7±11.3	60.0±14.2	0.197
LDL (mg/dl)	104.7±14.2	110.2±28.8	0.703
Baseline ALT (u/l)	22.4±6	24.5±9.2	0.614
ALT (> 30 u/l)	1(20%)	59(26%)	0.762
Total Bilirubin (mg/dl)	1.0±0.3	0.8±0.2	0.250
Platelets (10^3/μL)	197±19	220±49	0.301
Hemoglobin (Hb; g/dl)	15.1±1.4	14.2±1.5	0.183
AFP (α-fetal protein; ng/ml)	6.5±7.6	3.1±1.6	0.376
HBV DNA levels (log10 IU/ml)	2.3±1.5	3.0±1.2	0.158
HBV DNA > 2000 IU/ml	2 (40%)	105 (46.3%)	0.781
HBV DNA > 20000 IU/ml	0(0%)	38(16.7%)	0.317
qHBs Ag (log10 IU/ml)	0.3±1.4	2.5±1.0	0.005†
qHBsAg > 30 IU/ml (%)	1 (20%)	191 (84.1% %)	< 0.001†
qHBsAg > 100 IU/ml (%)	1 (20%)	173 (76.2%%)	0.004†
Stiffness (kPa)	5.1±2.8	4.5±1.2	0.301

*: Based on chi-square and independent samples t-tests.

^†^: P value < 0.05.

**Table 2:** Univariate and Multivariate Analyses of the Factors Associated With HBsAg Seroclearance.

Variables	Comparison	Univariate OR (95% CI)	P value	Multivariate OR (95% CI)	P value
Age (years)	Increase per one year	1.031 (0.952–1.118)	0.453		
Sex (Male:Female)	Female vs. Male	3.236 (0.361–28.571)	0.294		
DM (%)	+/-	0.047 (0.0–52261727)	0.774		
Hypertension (%)	+/-	0.041 (0.0–4866)	0.592		
BMI (kg/m2) (no. 5:225)	Increase per kg/m2	1.015 (0.773–1.331)	0.916		
Total cholesterol (mg/dL)	Decrease per one mg/dl	1.005 (0.974–1.036)	0.769		
Triglyceride (mg/dL)	Increase per one mg/dl	1.007 (0.998–1.015)	0.119		
HDL (mg/dl)	Decrease per one mg/dl	1.057 (0.968–1.152)	0.210		
LDL (mg/dl)	Decrease per one mg/dl	1.007 (0.969–1.046)	0.704		
Baseline ALT (u/l)	Decrease per one u/l	1.027 (0.926–1.140)	0.607		
ALT (> 30 u/l)	Less than 30 u/l	1.404 (0.156–12.5)	0.762		
Total Bilirubin (mg/dl)	Increase per one mg/dl	5.376(0.782–36.954)	0.087		
Platelets (10^3/μl)	Decrease per one 10^3/μL	1.122(0.908–1.386)	0.284		
Hemoglobin (g/dl)	Increase per one g/dl	1.555 (0.804–3.009)	0.19		
HBV DNA levels (log10 IU/ml)	Decrease per 1 log 10 IU/ml	1.739(0.805–3.759)	0.159		
HBV DNA > 2000 IU/ml	Less than 2000 IU/ml	1.305(0.218–7.812)	0.77		
qHBsAg (log10 IU/ML)	Decrease per 1 log 10IU/ml	2.257 (1.199–4.255)	0.012†		
qHBsAg > 30 IU/ml	Less than 30 IU/ml	19.607(2.207–166.666)	0.008†	19.607 (2.207–166.666)	0.008†
qHBsAg > 100 IU/ml	Less than 100 IU/ml	3.472 (1.161–10.387)	0.024†		
Stiffness (kPa)	Increase per one kPa	1.347 (0.770–2.357)	0.296		

^†^: P value < 0.05.

### Elevation of ALT above the ULN

Of the 235 HBeAg-negative patients, 15 patients (6.4%) experienced ALT elevations. These patients were predominantly male (male:female, 12:3, P = 0.051) and had higher baseline ALT levels (32.2 +/-12.2 vs. 23.9+/-8.8 u/l, P = 0.001), higher qHBsAg values (3.1+/-0.7 vs 2.4+/-1.1 log10 IU/ml, P = 0.003), and greater liver stiffness levels (5.1 +/-1.2 kPa vs 4.4 +/-1.3 kPa, P = 0.037, Table 3). Univariate analyses indicated that the factors that predicted ALT elevation were the baseline ALT level, liver stiffness, a HBV DNA > 20000 IU/ml and the qHBsAg value. The multivariate analysis revealed that the baseline ALT (OR 1.075/ IU/L, 95% CI 1.020–1.132, P = 0.006) and a qHBsAg above 1000 IU/ml (OR 3.7, 95% CI 1.1–12.4, P = 0.032) were significant predictors (Table 4). When limited to the 131 male patients, 12 patients (average age 46.7+/-9.5 years) experienced ALT elevation, and 119 patients (average age 48.9 +/- 10.8 years) did not experience ALT elevation. The multivariate analysis revealed that the baseline liver stiffness (OR 1.6, 95% CI 1.0–2.5, P = 0.031) and a qHBsAg above 1000 IU/ml (OR 10.4, 95% CI 2.1–52.4, P value 0.004) were independent risk factors for ALT elevation (Table 5).

**Table 3:** Basic characteristics of patients with and without ALT elevations above the upper limit of normal.

Group	ALT Elevation	Non-Elevation	P value*
Patient Number	15	220	
Age (years)	46.5±9.2	48.1±10.8	0.571
Sex (Male:Female)	12:03	119:101	0.051
Genotype (B, C, unknown)	11:03:01	162:47:11	0.957
DM (%)	0 (0%)	8 (3.6%)	0.452
Hypertension (%)	4(26.7%)	24(10.9%)	0.068
BMI (kg/m2)	24.5±3.6	23.3±3.1	0.171
BMI (kg/m2) (23 ≥)	11(73.3%)	111(50.9%)	0.171
Total cholesterol (mg/dl)	191.8±26.4	189.4±34	0.792
Triglyceride (mg/dl)	89.9±35.8	97.5±60.9	0.634
HDL (mg/dl)	58.2±12.5	59.8±14.3	0.674
LDL (mg/dl)	115.5±25.8	109.9±28.8	0.463
Baseline ALT (u/l)	32.2±12.2	23.96±8.8	0.001†
Baseline ALT (> 30 u/l)	7(46.7%)	54(24.5%)	0.059
Total Bilirubin (T-Bil; mg/dl)	0.8±0.3	0.8±0.3	0.828
Platelets (10^3/μL)	221±54	219±48	0.852
AFP (α-fetal protein; ng/ml)	3.2±1.1	3.1±2.0	0.882
HBV DNA levels (log10 IU/ml)	3.3±1.5	3.0±1.2	0.357
HBV DNA > 2000 IU/ml	8(53.3%)	99(45%)	0.531
HBV DNA > 20000 IU/ml	6(49%)	32(14.5%)	0.010†
HBs Ag titer (log10 IU/ml)	3.1±0.7	2.4±1.1	0.003†
qHBsAg > 1000 IU/ml (%)	11 (73.3%)	85(38.6%)	0.008†
Stiffness (kPa)	5.1±1.2	4.4±1.3	0.037†
Stiffness (>5 kPa)(%)	6(40%)	42(19.4%)	0.056
HBsAg Seroclearance during follow-up (%)	0 (0%)	8(3.6%)	0.452

*: Based on chi-square and independent samples t-tests.

^†^: P value < 0.05.

**Table 4:** Univariate and Multivariate analyses of the Factors Associated with ALT elevation above the upper limit of normal.

Variables	Comparison	Univariate OR (95% CI)	P value	Multivariate OR (95% CI)	P value
Age (years)	Decrease per one year	1.014 (0.965–1.066)	0.57		
Sex (Male:Female)	Male vs. Female	3.389 (0.931–1.234)	0.064		
BMI (kg/m2)	Increase 1 kg/m2	1.116 (0.953–1.307)	0.173		
BMI (kg/m2) (23 ≥)	Above 23 kg/m2	2.651(0.819–8.582)	0.104		
Total cholesterol (mg/dL)	Increase 1 mg/dl	1.002(0.987–1.018)	0.791		
Triglyceride (mg/dL)	Increase 1 mg/dl	0.997(0.987–1.008)	0.633		
HDL (mg/dL)	Increase 1 mg/dl	0.992(0.955–1.030)	0.672		
LDL (mg/dL)	Increase 1 mg/dl	1.006(0.990–1.023)	0.461		
Baseline ALT (u/l)	Increase 1 μ/l	1.084(1.030–1.140)	0.002†	1.075 (1.020–1.132)	0.006†
Baseline ALT (> 30 u/l)	Above 30 u/l	2.690(0.932–7.762)	0.067		
Total Bilirubin (T-Bil; mg/dL)	Increase per one mg/dl	1.209 (0.221–6.605)	0.827		
Platelets (10^3/μL)	Increase per one 10^3/μL	1.010 (0.909–1.122)	0.851		
AFP (α-fetal protein; ng/ml)	Increase per one ng/ml	1.019(0.797–1.303)	0.881		
DNA (log 10 IU/ml)	Increase per 1 log 10 IU/ml	1.228(0.793–1.901)	0.357		
HBV DNA > 2000 IU/ml	Above 2000 IU/ml	1.397(0.489–3.986)	0.532		
HBV DNA > 20000 IU/ml	DNA > 20000 IU/ml	3.917 (1.305–11.753)	0.015†		
qHBsAg (log 10 IU/ml)	Increase per 1 log 10 IU/ml	2.248 (1.089–4.643)	0.029†		
qHBsAg > 1000 IU/ml	qHBsAg > 1000 IU/ml	4.368(1.347–14.157)	0.014†	3.718 (1.119–12.359)	0.032†
Stiffness (kPa)	Increase per one kPa	1.431 (1.012–1.982)	0.043†		
Stiffness (>5 kPa)	Above > 5 kPa	2.778(0.937–8.233)	0.065		

^†^: P value < 0.05.

**Table 5:** Univariate and Multivariate analysis of the Factors Associated With ALT elevation above the upper limit of normal in the Males.

Variables	Comparison	Univariate OR (95% CI)	P value	Multivariate OR (95% CI)	P value
Age (years)	Decrease per one year	1.019 (0.964–1.077)	0.497		
BMI (kg/m2)	Increase 1 kg/m2	1.032 (0.857–1.242)	0.738		
BMI (kg/m2) (23 ≥)	Above 23 kg/m2	1.26(0.359–4.424)	0.718		
Total cholesterol (mg/dl)	Increase 1 mg/dl	1.001(0.985–1.018)	0.884		
Triglyceride (mg/dl)	Increase 1 mg/dl	0.985(0.967–1.004)	0.122		
HDL (mg/dl)	Increase 1 mg/dl	1.022(0.979–1.067)	0.322		
LDL (mg/dl)	Increase 1 mg/dl	1.004(0.986–1.022)	0.693		
Baseline ALT (U/L)	Increase per one U/l	1.069(1.002–1.142)	0.044†		
Baseline ALT (> 30 u/l)	Above 30 u/l	1.310(0.391–4.381)	0.662		
Total Bilirubin (T-Bil; mg/dl)	Decrease per one mg/dl	1.364 (0.198–9.345)	0.752		
Platelets (10^3/μl)	Increase per one 10^3/μl	1.020 (0.894–1.175)	0.726		
AFP (α-fetal protein; ng/ml)	Decrease per one ng/ml	1.018(0.768–1.347)	0.902		
DNA (log 10 IU/ml)	Increase per 1 log 10 IU/ml	1.509(0.853–2.667)	0.157		
HBV DNA > 2000 IU/ml	Above 2000 IU/ml	1.867(0.560–6.221)	0.309		
HBV DNA > 20000 IU/ml	DNA > 200000 IU/ml	4.952(1.392–17.615)	0.013†		
qHBsAg (log 10 IU/ml)	Increase per 1 log 10 IU/ml	2.971 (1.163–7.590)	0.023†		
qHBsAg > 1000 IU/ml	qHBsAg > 1000 IU/ml	10.256(2.143–49.087)	0.004†	10.447(2.081–52.441)	0.004†
Stiffness (kPa)	Increase per 1 kPa	1.556(1.066–2.272)	0.022†	1.606(1.043–2.473)	0.031†
Stiffness (>5 kPa)	> 5 kPa	4.008(1.146–14.023)	0.03†		

^†^: P value < 0.05.

## Discussion

HBsAg seroclearance is a landmark in the treatment of chronic HBV infection because hepatitis B is mainly immune-mediated, and the elimination of HBsAg is considered to be an indicator of the effective treatment of HBV infection. A lower HBsAg level may indicate better host immune control of HBV duplication and a decreased level of intrahepatic covalently closed circular DNA [10]. The annual HBsAg seroclearance rate of hepatitis B patients is between 0.5% and 2.26% per year depending on enrollment in treatment [11–13]. In a study conducted in Taiwan regarding spontaneous HBsAg seroconversion, the average follow-up period of hepatitis B patients was 7.4 years, and 18 patients of 3,000 cleared HBsAg with an annual rate of 0.6%. At 1 year after HBeAg seroconversion, low serum levels of HBsAg predicted the elimination of HBsAg in patients with genotype B or C infections [14]. A Korean study demonstrated that the overall annual HBsAg seroclearance rate was 1.8%, but the annual HBsAg seroclearance rate of the older-aged group was higher, i.e., the annual seroclearance rate was 2.69% among patients aged over 60, whereas the annual seroclearance rate was 1.91% among the patients aged between 40 and 59. The cumulative probabilities of HBsAg seroclearance were 1.2%, 3.8% and 8.7% for patients who were followed for 1, 3 and 5 years, respectively [15]. The annual HBsAg seroclearance rate in our study was 0.48%, i.e., 8 patients exhibited HBsAg seroclearance in the 1645 person-years study, and the rate was 0.3%, i.e., 5 patients exhibited HBsAg seroclearance in the 1624 person-years study. The overall cumulative HBsAg seroclearance rates were 0.4%, 1.3% and 2.3% for the patients who were followed up for 1, 3 and 5 years, respectively. In the present study, the HBsAg seroclearance rate was lower than that in the aforementioned Korean study. In the Korean study, the patients were all genotype C, and approximately 20% of them were treated with an antiviral agent or interferon; however, in our study, the patients were all treatment-naïve, and genotype B was predominant.

Given that there is no evidence of liver cirrhosis or HCV/HDV super infection and the subject’s age is <50 years at the time of the HBsAg loss, there is a minimal risk of HCC development [16]. The REVEAL-HBV study demonstrated that HBsAg seroclearance is significantly associated with increased age, a low serum HBV DNA level (100000 vs ≧ 100000 copies/mL) and a high body mass index (30 vs 30 kg/m2) [17]. In the natural course of chronic HBV infection, a lower HBV DNA level has been reported to be important in determining the subsequent elimination of HBsAg, and the HBV DNA levels in the sera of 95.8% of patients have been found to be undetectable before the elimination of HBsAg [18]. The HBsAg level was a better indicator than the HBV DNA level regarding the prediction of HBsAg elimination in patients who were followed for 6 years. Comparison of the patients with HBsAg levels ≧ 1000 IU/ml with those with HBsAg levels that were 100~999 and < 100 IU/ml revealed that the hazard ratios were 4.4 (95% CI, 1.1–17) and 24.3 (8.7–67.5), respectively, which indicated that the HBsAg loss rate of the latter group was higher. [14]. In a longitudinal study of 117 patients performed in Hong Kong, during the natural course of chronic hepatitis B, a HBsAg reduction > 1 log10 IU/ml was associated with greater viral suppression [19]. Our study revealed that the baseline HBV DNA level had nothing to do with HBsAg seroclearance, and only the qHBsAg was associated with HBsAg seroclearance; at the cutoff of qHBsAg 30 IU/ml, the HR was 19.230, and 95% CI was 2.2–166.7. There was no significant relationship between genotype and HBsAg seroclearance in our study. This study suggested the existence of an uncoupled immune effect on HBsAg production and viral duplication. However, a low qHBsAg level might suggest a more complete immune clearance than a low HBV DNA level.

At present, there is little data available for predicting spontaneous ALT elevation above the ULN in asymptomatic chronic HBV-infected patients with persistently normal ALT. In a recent study performed in India with patients with HBeAg-negative chronic hepatitis B who were yet asymptomatic and had normal ALT levels, the factors that were found to be predictive of the occurrence of spontaneous ALT flares were the male gender, the presence of precore mutants and an age equal to or above 30 years old at baseline. ALT flares were defined as elevations in the serum ALT level to above 2 times the ULN in combination with an HBV DNA level ≧105 copies/ml or a 100-fold rise in the HBV DNA from the previous baseline level. The rate of ALT flares was approximately 4.3% per year. The examined patients were predominantly genotypes D and A [20]. In a prospective study performed in Canada, ALT elevation was defined as a change from a normal ALT (ALT ≦ 40 IU/ml) to an elevated ALT (ALT > 40 IU/ml). This study followed 37 HBeAg-negative patients with normal ALT levels at baseline for a median of 3 years (0.67–4 years), and their baseline HBV DNA levels were found to be highly predictive of future ALT elevations above the ULN [21]. In contrast, a study of chronic hepatitis B among Asian American patients found that the ALT levels were normal (genotype B or C), and there were no strong associations of ALT flares with any of the assessed clinical factors, which included an age ≧ 50, gender, ALT at biopsy ≧ 1/2 ULN, fibrosis stage > 1, inflammation grade > 1, HBV DNA ≧ 100000 IU/ml or ≧ 20000 IU/ml, HBeAg positivity, HBV PC mutation, HBV BCP mutation, all combinations of PC and BCP mutations, and genotypes B or C. However, this study of Asian Americans did not test qHBsAg [22]. In our study, the annual rate of spontaneous ALT elevation was 0.9%, and the qHBsAg played a more important role in predicting ALT elevations. According to the medical care guide of the Taiwan National Health Insurance Administration, in the patient group with ALT elevations above the ULN, 7 of 15 patients (46.7%) would receive nucleoside/nucleotide analogues.

Arena et al. demonstrated that there is a strong association between liver stiffness and serum aminotransferase level [23]. In CHB patients with ALT flares, liver stiffness increases and returns to normal levels after 6 months [24]. A recent meta-analysis demonstrated that the FibroTest has good accuracy in terms of the identification of HBV-associated significant fibrosis and cirrhosis [25]. Regarding the prediction of ALT elevations above the ULN, the patients were further divided into male and female groups because different etiologies may be present in the different genders. However, there were only 3 females, so it was not possible to statistically analyze the females, and we only analyzed the male patients in the subgroup analysis. Baseline liver stiffness was correlated with future ALT elevation in our cohort. Across all patients, liver stiffness was only significant in the univariate analysis. However, in the subgroup analysis of the male patients, liver stiffness was significant in both univariate and multivariate analyses. We also analyzed the fibrosis-4 score (FIB-4) and AST to platelet ratio index (APRI). However, there was no significant relationship between these scores and ALT elevation.

qHBsAg provides not only a very useful means to identify the HBV-related cause of liver damage in HBsAg carriers but also it is the most important predictor of clinically relevant outcomes associated with HBV-induced liver disease. In a prospective study performed in France, reactivation of hepatitis among HBeAg-negative asymptomatic patients could be predicted by a combination of HBsAg > 1000 IU/ml and HBV-DNA > 200 IU/ml (92% sensitivity and 96% negative predictive value) [26]. Tseng TC et al. showed that HBsAg, ALT and age could be used as predictors for hepatocellular carcinoma in HBeAg negative patients with HBV DNA < 2000 IU/ml. This study revealed higher risk of HCC in patients with HBsAg ≥1000 IU/ml compared with those <1000 IU/ml (adjusted hazard ratio 13.7, 95% confidence interval: 4.8–39.3) [27]. Another prospective study from Korea also demonstrated that combined HBsAg and HBV DNA (> 850 IU/ml and 850 IU/ml, respectively) could predict the reactivation of HBV with 84.6% diagnostic accuracy [28]. One recent study showed that combined HBV DNA with qHBsAg or liver stiffness measurement could correctly identify inactive carrier at a single time point [29]. Our study emphasizes the influence of single baseline ALT, qHBsAg and liver stiffness measurement on natural history of asymptomatic hepatitis B infected patients. However, our study has some limitations. This study conducted only on a small sample size of population with relatively short duration of follow-up. We used the same level of ALT normal cutoff value (40 IU/ml) for both genders (2016 American Association for the Study of Liver Disease Guideline: normal ALT levels are <30 IU/ml for males and <10 IU/ml for female) because we use the same normal ALT cutoff value in real-world clinical practice in Taiwan.

In conclusion, our study demonstrated that a single baseline qHBsAg is an important factor for future ALT elevations above the ULN and qHBsAg seroclearance. Very few studies have used baseline liver stiffness to predict future ALT elevations. Currently, no simple and readily available markers are able to accurately predict biochemical elevations in HBV-infected patients. Using the qHBsAg, liver stiffness and HBV DNA, we have demonstrated a new perspective for predicting the natural course of HBeAg-negative patients with persistently normal liver function.

## Abbreviations

AFP: alpha fetoprotein
ALT: alanine transaminase
AST: aspartate transaminase
BMI: body mass index
HBV DNA: hepatitis B virus DNA
qHBsAg: quantitative HBsAg
ULN: upper limit normal
